# Supplementary material for: Development and Validation of the HIV-CARDIO-PREDICT Score to Estimate the Risk of Cardiovascular Events in HIV-Infected Patients
Source: Cells. 2023 Feb 5;12(4):523. doi: 10.3390/cells12040523 (PMC9953852; doi:10.3390/cells12040523)
Supplement: Supplementary file 1 [file cells-12-00523-s001.zip › cells-2106938-supplementary.pdf]

Table S1. Demographic, Clinical and Laboratory Characteristics of Patients Included in the Validation Cohort (N=383).

Patient Characteristics	
**Age (mean+/- SD)**	52.9 +/- 10.9
**Age at HIV diagnosis** (mean+/- SD)	34.0 +/- 13.7
**Gender**	
Male (%)	203 (53.5)
Female (%)	178 (46.5)
**Race**	
White (%)	150 (39.1)
African- American (%)	180 (46.9)
Hispanic/Latino (%)	50 (13.1)
Asian (%)	3 (7.8)
**Hypertension (%)**	91 (23.8)
**Hyperlipidemia (%)**	67 (17.5)
**Chronic Kidney Disease (%)**	101 (26.3)
**Diabetes Mellitus (%)**	(33.4)
**Hepatitis C**	64 (16.7)
**Smoking**	124 (32.3)
**Substance use disorder**	115 (30.0)
**Alcohol use disorder**	88 (22.9)
**Cardiovascular Events.**	**NO. OF PATIENTS (%)**
Sudden Cardiac Death	5 (1.3)
Hospitalization for Unstable Angina	21 (5.5)
Myocardial Infarction	26 (6.8)
Stroke	10 (2.6)
TIA	10 (2.6)
Carotid endarterectomy	6 (1.6)
CABG	11 (2.9)
**Total**	**89 (23.2)**
**CD4 at HIV diagnosis (mean, SD)**	**127 +/-88**
**CD4% at HIV diagnosis** (mean, SD)	10.1 +/-7.9
**CD4 nadir** (mean, SD)	86.4 +/-51.3
**CD4% nadir** (mean, SD)	8.2 +/-3.5
**Peak HIV Viral Load** (mean, SD)	380,774 +/-718,128
**HIV Viral Load at HIV diagnosis** (mean, SD)	299,291 +/-599,159
**Months to control HIV** (mean, SD)	5.5 (5.1)
**Months from HIV diagnosis to CVD event** (mean, SD)	79.3 (36.1)
**No. regimens to control HIV** (median, range)	1 (1-4)
**Adherence to ARVs** (median, range)	3 (1-4)
**Abacavir>6 months** (%)	61 (15.6)
